# Challenges and opportunities in understanding microbial communities with metagenome assembly (accompanied by IPython Notebook tutorial)

**DOI:** 10.3389/fmicb.2015.00678

**Published:** 2015-07-09

**Authors:** Adina Howe, Patrick S. G. Chain

**Affiliations:** ^1^GERMS Laboratory, Department of Agricultural and Biosystems Engineering, Iowa State University, Ames, IA, USA; ^2^Bioinformatics and Analytics Team, Bioscience Division, Los Alamos National Laboratory, Los Alamos, NM, USA

**Keywords:** metagenomes, assembly, review, challenges, tutorial

## Abstract

Metagenomic investigations hold great promise for informing the genetics, physiology, and ecology of environmental microorganisms. Current challenges for metagenomic analysis are related to our ability to connect the dots between sequencing reads, their population of origin, and their encoding functions. Assembly-based methods reduce dataset size by extending overlapping reads into larger contiguous sequences (contigs), providing contextual information for genetic sequences that does not rely on existing references. These methods, however, tend to be computationally intensive and are again challenged by sequencing errors as well as by genomic repeats While numerous tools have been developed based on these methodological concepts, they present confounding choices and training requirements to metagenomic investigators. To help with accessibility to assembly tools, this review also includes an IPython Notebook metagenomic assembly tutorial. This tutorial has instructions for execution any operating system using Amazon Elastic Cloud Compute and guides users through downloading, assembly, and mapping reads to contigs of a mock microbiome metagenome. Despite its challenges, metagenomic analysis has already revealed novel insights into many environments on Earth. As software, training, and data continue to emerge, metagenomic data access and its discoveries will to grow.

## Overview

The application of high throughput sequencing technologies for environmental microbiology is arguably as transformative as the invention of the microscope. When we began to *see* previously invisible microorganisms, we discovered the vast number of microbes in our environments. These observations significantly expanded the scope of microbiology as we began to have a better sense of the diversity of organisms outside of what we could grow in the laboratory. Presently, with sequencing technologies, we now *read* the genetic code of microorganisms, assembling microbial genomes without the need to even culture them, and in some cases providing clues as to how to culture them. This accessibility to genes has allowed us to investigate microorganisms and their predicted functional profiles in increasingly complex natural environments through approaches like metagenomics. In this review, we discuss how sequencing technologies can help us understand microbial communities and the challenges and opportunities involved in analyzing these very large datasets with metagenome assembly.

## Metagenomic Assembly

In analyzing microbes using genomics, one of the earliest forms of analysis involved genome assembly. Note that in this review, we use the phrase assembly to refer to *de novo* assembly, or the assembly of contigs without the use of previous references. From even the early days in sequencing, genome assembly has been a revered subspecialty in bioinformatics. Assembly began as an extension of local sequence alignments, where each sequencing read was compared with all other reads, followed by the subsequent assembly of the highest scoring pairs, essentially identifying overlapping sequences for extension into longer contiguous sequences, or contigs. These assemblers were developed for the then-standard Sanger sequencing technology. They were effective at retroactive correction of assembly errors, using the long, accurate Sanger read lengths for decision making with regards to variant calls and conflicts in read mate pairs that indicate possible chimeras or rearrangements (Dear and Staden, 1991; Lawrence et al., 1994; Myers, 1995; Bonfield and Whitwham, 2010).

The advent of next generation sequencing (NGS) technologies changed the type of sequencing data available to microbiologists and also expanded the types of questions that could be asked of sequencing. NGS reads are much cheaper than Sanger reads but are also much shorter in length (e.g., ∼100–250 bp). Assembly of NGS short read data is hampered both by the length of reads and the large number of reads that typically exceed by one or more orders of magnitude the number of reads that would be needed for the same project using Sanger sequencing. While fold coverage necessary for adequate assembly with Sanger data approached 10-fold coverage, with short-read technologies such as Illumina, the fold coverage needed for adequate assembly is generally 100-fold or greater (Sims et al., 2014). The number of read-to-read comparisons and the storing of this information quickly exceed the memory available on even very large memory machines. A series of more memory efficient methods based on *de Bruijn* graphs have been developed to tackle this assembly problem (Pevzner et al., 2001) and reviewed in (Pop, 2009; Miller et al., 2010).

Due to the increased cost-effectiveness, and to a lesser extent, the throughput of the newer, next-generation sequencing platforms, the number of shotgun metagenome projects in the microbiology field has surged. Today, thousands of projects are underway, exploring systems of low complexity, such as acid mine drainage (Tyson et al., 2004), ocean oil spills (Mason et al., 2012), and deep sea hydrothermal vents (Xie et al., 2011), to those of extreme complexity. In complex environments, metagenomes require deep sequencing for assembly; current sequencing efforts (less than 1 Tbp per sample) in soils and sediments resulting in less than half of the reads incorporated into assembled contigs (Luo et al., 2012; Howe et al., 2014) suggest that these environments contain very high diversity. While the specific goals of all these projects vary, most initial questions revolve around the characterization of functional and taxonomic composition. While there have been many recent advances in examining these questions using read-based approaches (Segata et al., 2012; Wood and Salzberg, 2014; Freitas et al., 2015), these are limited to supervised approaches, meaning that a limiting factor is the presence of an available database with appropriate reference genomes. For many of the ecosystems explored using metagenomics, there is a gross lack of high quality reference genomes. Without sufficiently similar references for dominant organisms in a sample, metagenome assembly is an approach that can provide greater insight into the community by delivering longer, contiguous sequences that can subsequently be investigated using more traditional approaches for classification of taxonomy and function. These contigs can sometimes approach the size of an entire genome, possibly linking functional genes to phylogenetic markers and allowing a more comprehensive reconstruction of the metabolic potential of a particular genome (Albertsen et al., 2013; Sharon et al., 2013; Wrighton et al., 2014).

## Current Challenges with Metagenome Assemblies

While the throughput of sequencers seems astronomical compared with a decade ago, it can still be difficult to have sufficient sequence representation from the large number of different organisms that can be found in many ecosystems. Due to variable relative abundance of different community members within a population, some genomes may be covered many thousands of times while others are only covered by a handful of sequencing reads or none at all. Some communities may even be sufficiently diverse that no member is represented very highly. Because any assembly of sequence data requires overlaps among reads, assembly of the less dominant members of a community may require additional sequencing.

These considerations, along with the cost, often dictate the level of sequencing effort dedicated to a project. The most prominent sequencing platforms currently used for metagenomes include ones that produces millions to billions of short (<300 bp) reads (e.g., Illumina sequencing platforms). Estimations of community diversity often precede metagenomic sequencing efforts. While these efforts (often using rRNA gene amplicon analysis) can be revealing for community studies by themselves, they can be inaccurate when it comes to strain-level diversification or population heterogeneity. For example, while some dominant rRNA members may be clonal in origin, others rRNA sequences may represent a broader diversity of genotypes.

Another challenge for metagenomic assembly is that despite the improvements in assembly algorithms and the advancement of computer hardware technology, assembly of such abundant, complex data can often overwhelm any given computer’s memory constraints. This issue is contributed to by the natural diversity of the community and the variants found within the population and is further exacerbated by sequencing errors that are present (even at very low levels) within the sequencing data.

## Strategies for Metagenome Assembly

There are an increasing number of assembly programs focused on the issue of metagenome assembly (Peng et al., 2011; Namiki et al., 2012; Li et al., 2015), most of which are based on *de Bruijn* graph assembly, that involves deconstructing the short reads into ever shorter *k*-mers of length k, finding overlaps of k-1, and traversing through the graph of *k*-mers/overlaps. There are a number of areas where metagenome assembly efforts have focused on improving. Some methods try to address the memory constraints in generating large assembly graphs, generally using a divide and conquer strategy. Other assemblers try to improve the ability to handle minor variants (or sequence errors) within otherwise identical *k*-mers by weighting *k*-mers by frequency or by collapsing paths depending on connectivity (e.g., bifurcating and rejoining paths). Other methods try to tackle some of the many complications that occur with the presence of genomes with high variations in abundance, for example by iterating over a series of different *k*-mer sizes. The length of the *k*-mer defines two things: 1) the overlap size needed among *k*-mers to allow assembly of two *k*-mers, and 2) the size of the repeat that can be resolved by the *k*-mer. Given sufficient coverage, longer *k*-mers will provide a simpler graph and a more robust assembly since repeats smaller than size k will be resolved within the graph. However, for organisms of lower abundance (i.e., genomes of lower coverage), the chance of sequencing overlapping regions (of size *k*) of the genome is also decreased (with longer *k* length), dictating the lower bound of organism abundance that can be assembled.

Because *de Bruijn* graph assembly is based on the smaller *k*-mer lengths and not on full read lengths, the smallest contigs are generally of size k+1, and it is possible to generate contigs from the graph that are not reflected by any read. If this was not already complicated, because of the highly conserved nature of functional features (homologous sequences) within disparate genomes, e.g., multiple copies of rRNA gene sequences, assemblers can generate chimeric contigs at any *k*-mer that is shared among two genomes (or within a genome). After assembly, contigs with minimal or no read coverage can be removed, and some of the chimeras can be resolved using paired-end reads if available. While these and other metagenome assembly issues can be somewhat addressed post-assembly, specialized tools are not yet available that address all of them. An alternative strategy for assembly of metagenomes includes using different algorithms that use reference genomes or genes for more specialized, targeted assembly (Boisvert et al., 2012).

## Accessibility to Metagenome Assembly

The challenges that face most scientists when confronted with metagenome assembly appear daunting: a wide array of assembly tools, each with their own strengths and weaknesses, and none ideal for any given metagenomic community of varying diversity, nor tailored to function within any given computational environment. In addition, this can become substantially more complex if using multiple technologies with differing error models, read lengths, and amounts of data since most bioinformatics tools are truly developed for highly specific data types.

Further exacerbating the situation is that most of these tools (especially newer ones) require knowledge of executing a command in a Unix environment. This obstacle, mainly the lack of individuals cross-trained in microbiology and practical bioinformatics is arguably one of the largest facing the field. Knowledge of the specific questions being asked of a sequencing dataset, the opportunities and limitations of an experiment, and the skills to effectively analyze these datasets can ensure that the data and algorithms used are appropriate for the question. While the number of microbiologists with bioinformatics skills is increasing, it is not yet commonplace, and sequencing is increasingly prevalent in most areas of biology and has already been declared democratized by a number of groups (Kumar et al., 2013; Koren et al., 2014; Meijueiro et al., 2014). As evident from the challenges above for metagenome assembly, even within the area of bioinformatics, there can be many subspecialties, each requiring a level of sophistication often beyond the average microbiologist. In an effort to make available some of the skills needed for metagenome analysis, including metagenome assembly, this review includes a tutorial on some of the steps for analyzing a simulated mock metagenome from the Human Microbiome Project.^1^ Given the challenges of accessibility to computational resources, this tutorial has been designed for implementation on rentable cloud computing.^2^ We also note that there are a number of challenges in metagenomics, and in this review, we focus on challenges facing individuals whose goal is to analyze a community using metagenome assembly. However, it is also important to consider that many other questions can be asked using a metagenome without specifically requiring an assembly (reviewed in, Sharpton, 2014), such as aligning reads to known references (reviewed in (Trapnell and Salzberg, 2009; Li and Homer, 2010; Fonseca et al., 2012) and read-based functional annotations (reviewed in, De Filippo et al., 2012; Prakash and Taylor, 2012).

### Conflict of Interest Statement

The authors declare that the research was conducted in the absence of any commercial or financial relationships that could be construed as a potential conflict of interest.

## Footnotes

1 http://hmpdacc.org/

2 http://nbviewer.ipython.org/github/germs-lab/frontiers-review-2015/blob/master/frontiers-nb-2015.ipynb

## References

[B1] Albertsen M. Hugenholtz P. Skarshewski A. Nielsen K. L. Tyson G. W. Nielsen P. H. (2013). Genome sequences of rare, uncultured bacteria obtained by differential coverage binning of multiple metagenomes. Nat. Biotechnol. 31, 533–538. 10.1038/nbt.2579 23707974

[B2] Boisvert S. Raymond F. Godzaridis E. Laviolette F. Corbeil J. (2012). Ray Meta: scalable *de novo* metagenome assembly and profiling. Genome Biol. 13, R122. 10.1186/gb-2012-13-12-r122 23259615 PMC4056372

[B3] Bonfield J. K. Whitwham A. (2010). Gap5–editing the billion fragment sequence assembly. Bioinformatics 26, 1699–1703. 10.1093/bioinformatics/btq268 20513662 PMC2894512

[B4] Dear S. Staden R. (1991). A sequence assembly and editing program for efficient management of large projects. Nucleic Acids Res. 19, 3907–3911. 10.1093/nar/19.14.3907 1861983 PMC328482

[B5] De Filippo C. Ramazzotti M. Fontana P. Cavalieri D. (2012). Bioinformatic approaches for functional annotation and pathway inference in metagenomics data. Brief. Bioinform. 13, 696–710. 10.1093/bib/bbs070 23175748 PMC3505041

[B6] Fonseca N. A. Rung J. Brazma A. Marioni J. C. (2012). Tools for mapping high-throughput sequencing data. Bioinformatics 28, 3169–3177. 10.1093/bioinformatics/bts605 23060614

[B7] Freitas T. A. K. Li P. E. Scholz M. B. Chain P. S. (2015). Accurate read-based metagenome characterization using a hierarchical suite of unique signatures. Nucleic Acids Res. 43, e69. 10.1093/nar/gkv180 25765641 PMC4446416

[B8] Howe A. C. Jansson J. K. Malfatti S. A. Tringe S. G. Tiedje J. M. Brown C. T. (2014). Tackling soil diversity with the assembly of large, complex metagenomes. Proc. Natl. Acad. Sci. U.S.A. 111, 4904–4909. 10.1073/pnas.1402564111 24632729 PMC3977251

[B9] Koren S. Treangen T. J. Hill C. M. Pop M. Phillippy A. M. (2014). Automated ensemble assembly and validation of microbial genomes. BMC Bioinform. 15:126. 10.1101/002469 24884846 PMC4030574

[B10] Kumar S. Jones M. Koutsovoulos G. Clarke M. Blaxter M. (2013). Blobology: exploring raw genome data for contaminants, symbionts and parasites using taxon-annotated GC-coverage plots. Front. Genet. 4:237. 10.3389/fgene.2013.00237 24348509 PMC3843372

[B11] Lawrence C. Honda S. Parrott N. W. Flood T. C. Gu L. Zhang L. (1994). The genome reconstruction manager: a software environment for supporting high-throughput DNA sequencing. Genomics. 23, 192–201. 10.1006/geno.1994.1477 7829071

[B12] Li D. Liu C. M. Luo R. Sadakane K. Lam T. W. (2015). MEGAHIT: An ultra-fast single-node solution for large and complex metagenomics assembly via succinct de Bruijn graph. Bioinformatics 31, 1674–1676. 10.1093/bioinformatics/btv033 25609793

[B13] Li H. Homer N. (2010). A survey of sequence alignment algorithms for next-generation sequencing. Brief. Bioinform. 11, 473–483. 10.1093/bib/bbq015 20460430 PMC2943993

[B14] Luo C. Tsementzi D. Kyrpides N. C. Konstantinidis K. T. (2012). Individual genome assembly from complex community short-read metagenomic datasets. ISME J. 6, 898–901. 10.1038/ismej.2011.147 22030673 PMC3309356

[B15] Mason O. U. Hazen T. C. Borglin S. Chain P. S. Dubinsky E. A. Fortney J. L. (2012). Metagenome, metatranscriptome and single-cell sequencing reveal microbial response to Deepwater Horizon oil spill. ISME J. 6, 1715–1727. 10.1038/ismej.2012.59 22717885 PMC3498917

[B16] Meijueiro M. L. Santoyo F. Ramírez L. Pisabarro A. G. (2014). Transcriptome characteristics of filamentous fungi deduced using high-throughput analytical technologies. Brief. Funct. Genomics 13, 440–450. 10.1093/bfgp/elu033 25212483

[B17] Miller J. R. Koren S. Sutton G. (2010). Assembly algorithms for next-generation sequencing data. Presentation. Genomics 95, 315–327. 10.1016/j.ygeno.2010.03.001 20211242 PMC2874646

[B18] Myers E. W. (1995). Toward simplifying and accurately formulating fragment assembly. J. Comput. Biol. 2, 275–290. 10.1089/cmb.1995.2.275 7497129

[B19] Namiki T. Hachiya T. Tanaka H. Sakakibara Y. (2012). MetaVelvet: an extension of Velvet assembler to *de novo* metagenome assembly from short sequence reads. Nucleic Acids Res. 40, e155. 10.1093/nar/gks678 22821567 PMC3488206

[B20] Peng Y. Leung H. C. Yiu S. M. Chin F. Y. (2011). Meta-IDBA: a *de Novo* assembler for metagenomic data. Bioinformatics 27, i94–i101. 10.1093/bioinformatics/btr216 21685107 PMC3117360

[B21] Pevzner P. A. Tang H. Waterman M. S. (2001). An Eulerian path approach to DNA fragment assembly. Proc. Natl. Acad. Sci. U.S.A. 98, 9748–9753. 10.1073/pnas.171285098 11504945 PMC55524

[B22] Pop M. (2009). Genome assembly reborn: recent computational challenges. Brief. Bioinform. 10, 354–366. 10.1093/bib/bbp026 19482960 PMC2691937

[B23] Prakash T. Taylor T. D. (2012). Functional assignment of metagenomic data: challenges and applications. Brief. Bioinform. 13, 711–727. 10.1093/bib/bbs033 22772835 PMC3504928

[B24] Segata N. Waldron L. Ballarini A. Narasimhan V. Jousson O. Huttenhower C. (2012). Metagenomic microbial community profiling using unique clade-specific marker genes. Nat. Methods 9, 811–814. 10.1038/nmeth.2066 22688413 PMC3443552

[B25] Sharon I. Morowitz M. J. Thomas B. C. Costello E. K. Relman D. A. Banfield J. F. (2013). Time series community genomics analysis reveals rapid shifts in bacterial species, strains, and phage during infant gut colonization. Genome Res. 23, 111–120. 10.1101/gr.142315.112 22936250 PMC3530670

[B26] Sharpton T. J. (2014). An introduction to the analysis of shotgun metagenomic data. Front. Plant Sci. 5:209. 10.3389/fpls.2014.00209 24982662 PMC4059276

[B27] Sims D. Sudbery I. Ilott N. E. Heger A. Ponting C. P. (2014). Sequencing depth and coverage: key considerations in genomic analyses. Nat. Rev. Genet. 15, 121–132. 10.1038/nrg3642 24434847

[B28] Trapnell C. Salzberg S. L. (2009). How to map billions of short reads onto genomes. Nat. Biotechnol. 27, 455–457. 10.1038/nbt0509-455 19430453 PMC2836519

[B29] Tyson G. W. Chapman J. Hugenholtz P. Allen E. E. Ram R. J. Richardson P. M. (2004). Community structure and metabolism through reconstruction of microbial genomes from the environment. Nature 428, 37–43. 10.1038/nature02340 14961025

[B30] Wood D. E. Salzberg S. L. (2014). Kraken: ultrafast metagenomic sequence classification using exact alignments. Genome Biol. 15, R46. 10.1186/gb-2014-15-3-r46 24580807 PMC4053813

[B31] Wrighton K. C. Castelle C. J. Wilkins M. J. Hug L. Sharon I. Thomas B. C. (2014). Metabolic interdependencies between phylogenetically novel fermenters and respiratory organisms in an unconfined aquifer. ISME J. 8, 1452–1463. 10.1038/ismej.2013.249 24621521 PMC4069391

[B32] Xie W. Wang F. Guo L. Chen Z. Sievert S. M. Meng J. (2011). Comparative metagenomics of microbial communities inhabiting deep-sea hydrothermal vent chimneys with contrasting chemistries. ISME J. 5, 414–426. 10.1038/ismej.2010.144 20927138 PMC3105715

